# Pharmacokinetics and Tissue Distribution Study of Chlorogenic Acid from Lonicerae Japonicae Flos Following Oral Administrations in Rats

**DOI:** 10.1155/2014/979414

**Published:** 2014-07-21

**Authors:** Yulu Zhou, Ting Zhou, Qi Pei, Shikun Liu, Hong Yuan

**Affiliations:** ^1^Department of Pharmacy, The Third Xiangya Hospital, Central South University, Changsha, Hunan 410013, China; ^2^School of Pharmaceutical Sciences, Central South University, Changsha, Hunan 410013, China

## Abstract

Chlorogenic acid (ChA) is proposed as the major bioactive compounds of Lonicerae Japonicae Flos (LJF). Forty-two Wistar rats were randomly divided into seven groups to investigate the pharmacokinetics and tissue distribution of ChA, via oral administration of LJF extract, using ibuprofen as internal standard, employing a high performance liquid chromatography in conjunction with tandem mass spectrometry. Analytes were extracted from plasma samples and tissue homogenate by liquid–liquid extraction with acetonitrile, separated on a *C*_18_ column by linear gradient elution, and detected by electrospray ionization mass spectrometry in negative selected multiple reaction monitoring mode. Our results successfully demonstrate that the method has satisfactory selectivity, linearity, extraction recovery, matrix effect, precision, accuracy, and stability. Using noncompartment model to study pharmacokinetics, profile revealed that ChA was rapidly absorbed and eliminated. Tissue study indicated that the highest level was observed in liver, followed by kidney, lung, heart, and spleen. In conclusion, this method was suitable for the study on pharmacokinetics and tissue distribution of ChA after oral administration.

## 1. Introduction

**Figure 1:**
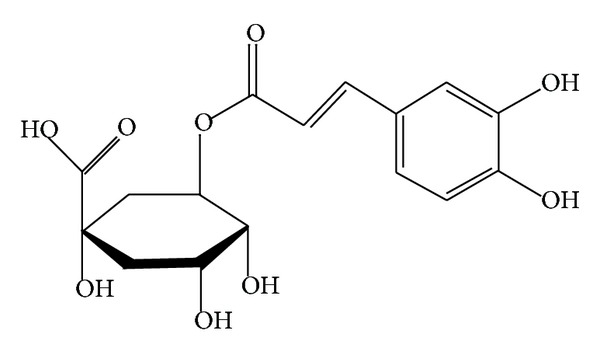
Chemical structures of chlorogenic acid.

Lonicerae Japonicae Flos (LJF) (*Lonicera japonica* Thunb), as a traditional Chinese medicine, was used widely in diseases such as wind-heat common cold and hot blood poison dysentery for its main property of clearing heat and detoxication [1]. The accumulated evidence has demonstrated that LJF has dozens of chemical components such as chlorogenic acid (ChA) (Figure 1), adinoside A, and stryspinoside [2, 3]. As the main active ingredient of LJF, ChA not only is the most abundant phenolic acid in LJF but also is usually used to control the quality of the LJF by its exact content [4]. ChA can prevent oxidation and microbial infection, protect cardiovascular system and liver, decrease blood pressure, and attenuate inflammation and pain [5, 6]. Furthermore, ChA can also inhibit the replication and viability of* Enterovirus* 71* in vitro* [7] and show effective activity of antibacterial and antibiofilm [8, 9]. Additionally, ChA can reduce liver inflammation and fibrosis through inhibition of toll-like receptor 4 signaling pathway [10], attenuate ventricular remodeling after myocardial infarction [11], relieve acute and inflammatory pain [12], and prevent from lens opacity and cytotoxicity in human lens epithelial cells [13]. Concerning the multiple biological activity of ChA, investigating the pharmacokinetic profile and tissue distribution of ChA is instant requirement for its clinic application.

In this study, we investigated firstly the pharmacokinetic and tissue distribution of ChA extract from LJF* in vivo* by using high performance liquid chromatography in conjunction with tandem mass spectrometry (HPLC-MS/MS). A rapid and sensitive HPLC-MS/MS method was developed and validated to describe the pharmacokinetics and tissue distribution of ChA after oral administration of LJF extract in rats.

## 2. Experiment

### 2.1. Materials and Reagents

LJF was bought in Changsha. The reference standards of ChA and ibuprofen were supplied by China National Institute for the Control of Pharmaceutical and Biological Products (Beijing, China). Acetonitrile (ACN) and methanol (MEOH), HPLC grade, were acquired from Merck (Darmstadt, Germany). Formic acid was of HPLC grade and purchased from ROE SCIENTIFIC INC. (Beijing, China).

### 2.2. Animals, Drug Administration, and Sampling

Wistar rats weighting 180–220 g, half male and half female, were purchased from Changsha Tianqing Biotechnology Limited Company (Changsha, China). The rats were housed for 1 week, room temperature (22 ± 2°C), relative humidity (45–60%), and 12 h dark/light cycle controlled facility with free access to food and tap water. In this study, 42 rats were randomly assigned to seven groups, and rats fasted with free access to water for 12 h before being dosed. LJF extract was dissolved with 20% PEG400 and administered orally at a dose of 400 mg/kg.

Blood samples and tissue samples were collected at 10, 30, 60, 120, 180, and 240 min after dose. Blood samples were put into heparinized microcentrifuge tubes, followed by centrifuging at approximately 11000 r/min for 5 min. The resulting plasma layers were separated and stored in microcentrifuge tubes at −80°C until the analysis. Tissue samples were weighed rapidly and put into normal saline solution to remove the blood by blotting on filter paper and were minced and homogenized with physiological saline solution (1 : 2, w/v) thoroughly in ice-bath. These tissue homogenates were stored at −80°C until the analysis.

### 2.3. Preparation of Calibration Standard and Quality Control Samples

The stock solution of ChA was prepared by accurately weighing 10.00 mg of ChA reference substance into 10 mL volumetric flasks and dissolving in MEOH to give a final concentration of 1.000 g/L, so did ibuprofen. The standard solutions of ChA with concentrations of 78.20, 156.30, 312.50, 625.00, 1250.00, 2500.00, 5000.00, and 10000.00 *μ*g/L were prepared by further dilution of the stock solution with MEOH. The working solutions of ibuprofen (5 mg/L) were obtained by dilution of the stock solution with MEOH. All the solutions were stored at 4°C and brought to room temperature before use.

To prepare the standard calibration samples, 20 *μ*L of ChA standard solutions was added to 200 *μ*L of controlled blank plasma. The mixture was vortex-mixed thoroughly to get the final standard concentrations of 7.820, 15.630, 31.250, 62.500, 125.000, 250.000, 500.000, and 1000.000 *μ*g/L. The quality control (QC) samples with the concentration of 15.630, 125.000, and 800.000 *μ*g/L were prepared by fortifying 20 *μ*L of ChA standard solution to 200 *μ*L of controlled blank plasma. These samples were stored at −20°C.

### 2.4. Sample Preparation

The preparation of LJF extract was operated as follows: 200 g of LJF with 2000 mL 85% ETOH was refluxed three times each for 2 hours. Subsequently, the solution was concentrated under reduced pressure and dried with microwave, yielding an extract with the content of 16.7% (ChA) detected by HPLC-MC/MC, which was much higher than the content of 2.51% (ChA) in LJF.

Twenty microliters of internal standard liquid (5 mg/L ibuprofen) which was added, during analysis, to 200 *μ*L of biosamples of tissue homogenate, plasma, standard calibration samples, or QC samples followed by addition of 20 *μ*L of internal standard liquid (5 mg/L ibuprofen), was added; then 400 *μ*L of acetonitrile was added to precipitate protein. The biological samples were swirled for 1 min and were centrifuged at 11000 r/min for 10 min at 4°C. The obtained supernatant was filtered via a 0.22 *μ*m filter membrane, and 5 *μ*L of supernatant was injected into the HPLC-MS for analysis.

### 2.5. Instrumentation and HPLC-MS/MS Conditions

The HPLC-MS/MS system consists of an UFLC-20A high performance liquid chromatograph (Shimadzu Corporation, Japan), including an autosampler and temperature controlled column compartment, and an API 4000 mass spectrometer/mass spectrometer (AB SCIEX, America) with an electrospray ionization source (ESI). The signal acquisition, peak integration, and concentration determination were performed using the Analyst 1.5.1 software, supplied by AB SCIEX (Boston, America), too.

Chromatographic separation was performed on guard column *C*_18_ column (3 *μ*m, 4 mm × 2.0 mm, Phenomenex, Torrance, CA, USA) and Luna *C*_18_ column (3 *μ*m, 50 mm × 2.0 mm, Phenomenex). The autosampler temperature was maintained at 25°C and the column at 4°C. A gradient of 0.01% formic acid in methanol (solvent A) and 0.01% formic acid in water (solvent B) was used as follows: 95% B at 0.00 to 0.50 mins, 20% B at 0.50 to 2.00 mins, and 95% B at 2.00 to 4.50 mins. The flow rate was 0.4 mL/min.

The mass spectrometer equipped with an ESI (in the positive mode) source was performed in negative ion MRM mode, set with the capillary voltage of 4500 V; the pressure of ion source gas (N_2_) 1 is 4.5 × 10^5^ Pa and 2 is 5.5 × 10^5^ Pa; the air curtain gas (N_2_) is 2.0 × 10^5^ Pa. The monitor ions of chlorogenic acid are from m/z 353.0 to m/z 191.1, declustering potential (DP) of 56 V, collision energy (CE) of 21 eV, while ibuprofen is from m/z 204.9 to m/z 161.0, DP of 53 V, CE of 11 eV.

### 2.6. Method Validation

#### 2.6.1. Selectivity

In order to investigate potential interference from endogenous compounds that could coelute with the analyte and the internal standard, 200 *μ*L of blank rat plasma from six different sources with or without standard solutions of ChA and ibuprofen and plasma samples was tested after the administration.

#### 2.6.2. Linearity and Lower Limit of Quantification (LLOQ)

Linearity of calibration curve was determined by plotting the peak area ratio (*y*) of ChA to internal standard versus the concentration (*x*) of ChA. Series calibration standards of plasma and different tissues were prepared as descried above for analysis. Results were fitted to linear regression analysis using 1/*x* as the weighting factor.

#### 2.6.3. Extraction Recovery and Matrix Effect

To calculate recovery, QC samples were analyzed at low (15.63 *μ*g/L), medium (125.00 *μ*g/L), and high (800.00 *μ*g/L) concentration in quintuplicate after the preparation method described above. The peak area of ChA (A1) and ibuprofen (A2) in plasma samples was noted. Besides, 20 *μ*L of internal standards as well as 600 *μ*L of mobile phases was added to each concentration of 200 *μ*L of QC samples followed by swirling for 30 sec. And 5 *μ*L of mix solution was taken for injection. The peak area of ChA (A1′) and ibuprofen (A2′) in blood samples was noted.

The recovery was calculated by using the following formula: ChA in standard plasma samples (%) = A1/A1′; ibuprofen in standard plasma samples (%) = A1/A2′.To calculate matrix effect (ME), 20 *μ*L of internal standards was added to each concentration of 200 *μ*L of QC samples; also, 600 *μ*L of blank plasma or mobile phases, swirled for 30 sec, and 5 *μ*L of mix solution were taken for injection. The peak area of ChA (A3, A4) and ibuprofen (A3′, A4′) in plasma samples was recorded. ME can be determined by using the following formula: ChA in standard plasma samples (%) = A3/A3′; ibuprofen in standard plasma samples (%) = A4/A4′.

#### 2.6.4. Accuracy and Precision

In order to determine the intraday accuracy and precision, five replications of all low, medium, and high concentration QC samples were performed on the same day and calculated each concentration of samples according to the calibration curve. The interday accuracy and precision were assessed by analyzing three batches on different days. The criteria for data acceptability are as follows: accuracy was determined by the ratio of calculated concentration and nominal concentration, precision was evaluated by relative standard derivative (RSD), and both accuracy and precision were within 15%.

#### 2.6.5. Stability

Freeze-thaw stability was determined by assessing the QC samples after three freeze and thaw cycles at room temperature and at −80°C. The short-term stability was evaluated by keeping the QC samples at room temperature for 3 h and 6 h. The postpreparative stability was conducted by reanalyzing the QC samples after 6 h and 12 h in the autosampler at 4°C. The long-term stability of ChA was determined by placing the QC samples at −80°C for 30 days. For all storage conditions, replications of all low, medium, and high concentration QC samples were analyzed after the operation and the experimental results were obtained through chromatographic area and compared with the nominal values.

## 3. Results 

### 3.1. Method Validation

#### 3.1.1. Selectivity

**Figure 2:**
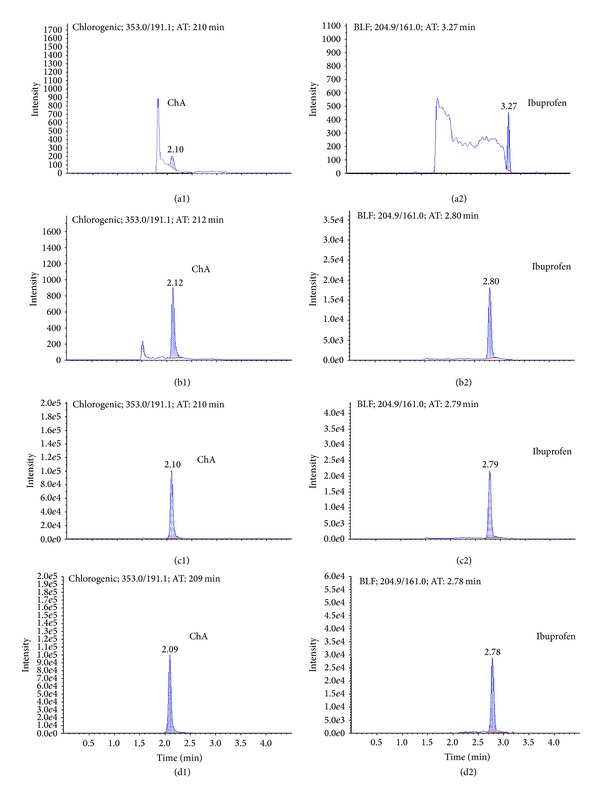
Representative mass spectrum of ChA and ibuprofen. (a1) Blank plasma sample of ChA. (a2) Blank plasma sample of ibuprofen. (b1) Standard calibration plasma sample with ChA at LLOQ level (7.820 *μ*g/L). (b2) Standard calibration plasma sample with ibuprofen at LLOQ level. (c1) Plasma sample after administration of LJF extract of ChA. (c2) Plasma sample after administration of LJF extract of ibuprofen. (d1) Liver sample of ChA. (d2) Liver sample of ibuprofen.

No interfering peaks were observed at the retention time of ChA (2.1 min) and internal standard (ibuprofen, 2.8 min) in all conditions (Figure 2).

#### 3.1.2. Linearity and Lower Limit of Quantification (LLOQ)

The back-calculated concentrations (mean ± SD) of ChA from the representative calibration standards by HPLC-MS/MS determination were within the acceptance limits. The correlation coefficient of calibration curve is larger than 0.99, suggesting a good linearity within the range from 7.820 *μ*g/L to 1000.000 *μ*g/L. The LLOQ of ChA is 7.820 *μ*g/L in plasma sample and tissues.

#### 3.1.3. Extraction Recovery and Matrix Effect

Extraction recovery and matrix effect were computed. The mean extraction recovery at three concentrations of ChA was 94.7% and of ibuprofen was 93.8%. The mean matrix effect at three concentrations of ChA was 96.7% and of ibuprofen was 94.9%.

#### 3.1.4. Accuracy and Precision

Accuracy and precision data for intra- and interday plasma samples were within the scope of the standard (Table 1). The intra- and interday mean accuracy were within 5.0%; the intra- and interday precision (RSD%) values were less than 8%.

#### 3.1.5. Stability

The stability data of ChA under four conditions are listed in Table 2. The ChA in plasma has been stable for 6 h at room temperature, for 12 h in autosampler, after 3 freeze-thaw cycles, and for 30 days stored at −80°C.

### 3.2. Pharmacokinetic Analysis

**Figure 3:**
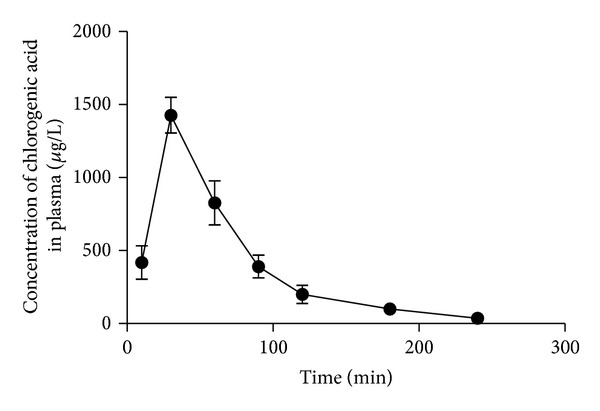
Plasma concentration-time profiles of ChA after oral administration. Each point represents the mean ± SD of 6 rats.

The pharmacokinetic analysis was processed with WinNonlin 6.1 software simulating data with noncompartmental model. Mean plasma concentration-time profile and the corresponding pharmacokinetic parameters of ChA after oral administration are shown in Figure 3 and Table 3. In this study, we found that ChA was absorbed and eliminated rapidly in rats, with low oral bioavailability. The half-lives of ChA in the plasma were about 0.8 h; the *t*_max⁡_ was 0.58 ± 0.13 h and *C*_max⁡_ was 1490 ± 0.16 ug/L.

### 3.3. Tissue Distribution Analysis

**Figure 4:**
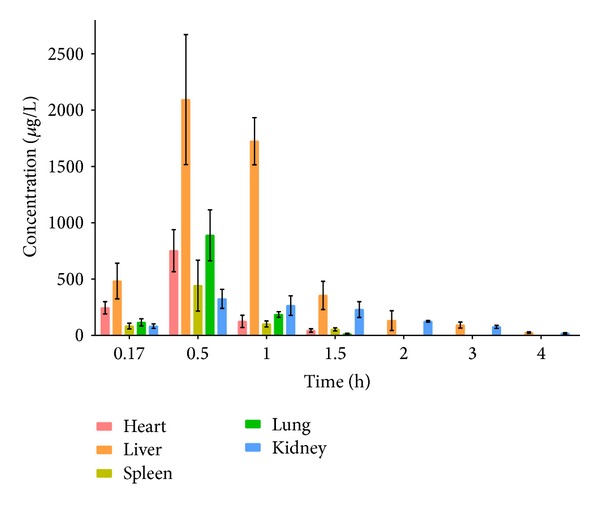
Tissue distribution of ChA in rats after oral administration. Each point represents the mean ± SD of 6 rats.

The concentration of ChA was determined in several organs, which indicated that ChA rapidly increased and then decreased accompanied by a wide distribution. Tissue distribution showed that the highest level was in the liver, followed by kidney, lung, heart, and spleen as shown in Figure 4. The concentrations in organs revealed that ChA was metabolized quickly and it almost cannot be detected in tissues after 4 h.

## 4. Discussion

Considering the low solubility of ChA, 20% PEG400 was selected as solubilization to improve the dissolving capacity of ChA. And, according to our preliminary experiments, ChA is mainly distributed to the most abundant blood-supply tissues, such as liver and kidney, which implied that the distribution of ChA might depend on the blood flow and perfusion rate of the organ. Then, we explored the biotransformation of primary ChA in liver and kidney. ChA decreased more rapidly in liver than that in kidney, which showed that liver played a more important role as compared to kidney. Furthermore, it can be inferred that ChA might target liver and induce a protective effect. Farrell et al. have studied the absorption and metabolism of ChA in cultured gastric epithelial monolayers [14], but cell experiment has its intrinsic limits. Pharmacokinetics of ChA extracted from Shuang-Huang-Lian [15], Yin-Huang granules [16], Aidi lyophilizer [17], or Daqingye [18] was also studied, while little in LJF. Other methods, such as HPLC [17, 19], LC-MS/MS [15], RP-HPLC [16], or HPLC­DAD [4], were used to detect ChA.

By using the animal experiment, we can explore multiple pharmacological effects of LJF extract (ChA) as possible. And HPLC-MS/MS applied in our study exhibited higher sensitivity than LC-MS/MS. As we know,* in vivo* study of pharmacokinetic and tissue distribution of LJF extract (ChA) is significantly meaningful. Pharmacokinetic study can contribute to better understand the efficacy and toxicity of ChA, furthermore, tissue distribution study is crucial to discover the main target sites and account for disposition [20]. So, we first found a HPLC-MS/MS method, which is simple, sensitive, and with a highly detective *C*_max⁡_ and short *t*_max⁡_.

Although we established this method and found some important meaningful knowledge, there are still some respects that need to ameliorate. For the pharmacokinetic, further experimental data from animals even human* in vivo *study is needed to be conducted. For the tissue distribution, we determined the concentration of ChA in each organ; the method which is used for quantification of ChA in tissues needs to be verified, just like the method that is applied in quantification of ChA in rat plasma.

## 5. Conclusion 

In conclusion, for the first time, a rapid, simple, and sensitive HPLC-MS/MS method was validated for the quantification of ChA in rat plasma and tissues. The specificity, linearity, extraction recovery, accuracy, and stability of the method were successfully established.

## Figures and Tables

**Table 1:** Accuracy and precision of ChA in plasma samples.

Added concentration (*μ*g/L)	Mean measured concentration (*μ*g/L)	Accuracy (%)	RSD (%)
Intraday (*n* = 5)			
15.63	15.70	100.4	6.4
125.00	127.60	102.1	7.1
800.00	839.00	104.9	7.6
Interday (*n* = 15)			
15.63	15.71	100.5	6.7
125.00	123.90	99.1	6.4
800.00	834.00	104.3	7.9

**Table 2:** The stability of plasma samples.

Stability type	Time and *n*	Added concentration (*μ*g/L)	Mean measured concentration (*μ*g/L)	Accuracy (%)	RSD (%)
Short-term stability	3 h (*n* = 3)	15.63	14.30	91.5	7.0
		125.00	131.4	105.1	5.7
		800.00	825.5	103.2	3.0
	6 h (*n* = 3)	15.63	14.22	91.0	6.8
		125.00	133.6	106.9	6.2
		800.00	806.5	100.8	5.4

Postpreparative stability	6 h (*n* = 5)	15.63	14.30	91.5	7.0
		125.00	131.4	105.1	5.7
		800.00	825.5	103.2	3.0
	12 h (*n* = 5)	15.63	15.252	97.6	9.5
		125.00	130.3	104.2	6.4
		800.00	842	105.3	7.1

Freeze-thaw stability	After 3 freeze-thaw cycles (*n* = 3)	15.63	13.74	87.9	7.7
		125.00	127.3	101.9	6.4
		800.00	771.3	96.4	4.6

Long-term stability	After 30 days (*n* = 3)	15.63	13.66	87.4	5.2
		125.00	122.1	97.7	3.1
		800.00	804.9	100.6	4.0

**Table 3:** Pharmacokinetic parameters of ChA in rat plasma after oral administration (*n* = 6).

Parameters	Value	Parameters	Value
*C*_max⁡_ (*μ*g/L)	1490.00 ± 160.00	*t*_1/2_ (h)	0.80 ± 0.54
*t*_max⁡_ (h)	0.58 ± 0.13	AUC_0→*t*_ (*μ*g∗h/L)	1700.00 ± 320.00
*V*/*F* (L)	266.85 ± 144.89	AUC_0→*∞*_ (*μ*g∗h/L)	1730.00 ± 330.00
CL/*F* (L/h)	238.53 ± 49.76	MRT_0→*t*_ (h)	1.07 ± 0.09
